# Does an additional structured information program during the intensive care unit stay reduce anxiety in ICU patients?: a multicenter randomized controlled trial

**DOI:** 10.1186/1471-2253-14-48

**Published:** 2014-06-28

**Authors:** Steffen Fleischer, Almuth Berg, Johann Behrens, Oliver Kuss, Ralf Becker, Annegret Horbach, Thomas R Neubert

**Affiliations:** 1 Institute of Health and Nursing Science, Medical Faculty, Martin-Luther-University Halle-Wittenberg, Halle, Germany; 2 Institute of Medical Epidemiology, Biostatistics, and Informatics, Medical Faculty, Martin-Luther-University Halle-Wittenberg, Halle, Germany; 3 Städtisches Klinikum München GmbH Akademie, Munich, Germany; 4 Sana Herzchirurgische Klinik Stuttgart, Stuttgart, Germany; 5 Department 4: Health and Social Work, University of Applied Sciences, Frankfurt/Main, Germany; 6 Hessian Institute of Nursing Research (HessIP), Franfurt/Main, Germany; 7 Department of Nursing Research, University Hospital Giessen and Marburg, Location Marburg, Germany; 8 Institute for Theoretical Surgery/Department of Visceral, Thoracic and Vascular Surgery, University Hospital Giessen and Marburg, Location Marburg, Germany

**Keywords:** Intensive care units, Critical care, Anxiety, Prevention & control, Nurse-patient relations, Information, Randomized controlled trial

## Abstract

**Background:**

Communication and information in order to reduce anxiety in the intensive care unit (ICU) has been described as area needing improvement. Therefore, the aim of this trial was to evaluate whether a structured information program that intensifies information given in standard care process reduces anxiety in ICU patients.

**Methods:**

Multicenter, two-armed, non-blinded, parallel-group randomized controlled trial in hospitals in the cities of Marburg, Halle, and Stuttgart (Germany). The trial was performed in cardiac surgery, general surgery, and internal medicine ICUs. Two-hundred and eleven elective and non-elective ICU patients were enrolled in the study (intervention group, n = 104; control group, n = 107). The experimental intervention comprised a single episode of structured oral information that was given in addition to standard care and covered two main parts: (1) A more standardized part about predefined ICU specific aspects – mainly procedural, sensory and coping information, and (2) an individualized part about fears and questions of the patient. The control group received a non-specific episodic conversation of similar length additional to standard care. Both conversations took place at the beginning of the ICU stay and lasted 10–15 minutes. Study nurses administered both interventions. The primary outcome ICU-related anxiety (CINT-Score, 0–100 pts., higher scores indicate higher anxiety) was assessed after admission to a regular ward.

**Results:**

The primary outcome could be measured in 82 intervention group participants and 90 control group participants resulting in mean values of 20.4 (SD 14.4) compared to 20.8 (SD 14.7) and a mean difference of −0.2 (CI 95% -4.5 to 4.1).

**Conclusions:**

A structured information intervention additional to standard care during ICU stay had no demonstrated additional benefit compared to an unspecific communication of similar duration. Reduction of anxiety in ICU patients will probably require more continuous approaches to information giving and communication.

**Trial registration:**

ClinicalTrials.gov NCT00764933.

## Background

Qualitative and quantitative studies report patients’ experience of intensive care unit (ICU) stay as highly stressful [1-5]. Psychological and physical stress in ICUs often is associated with higher levels of anxiety and feelings of uncertainty and helplessness [6,7]. Evidence suggests that such elevated and prolonged stress has negative consequences on health outcomes like delayed wound healing, susceptibility to respiratory infections, changed immune responses to vaccines [8], and is conceived as a risk factor of delirium [9]. Ineffective communication and lack of information further contribute to distress for patients [10-18]. International guidelines therefore emphasize the importance of effective communication for patient-centered care, especially with critically ill patients, but still there is little evidence on effective interventions [19,20]. This coincides with a recent Delphi study that rated priorities of ICU-related research in the domain of patient wellbeing [21]: Strategies to reduce stress and anxiety, and strategies to improve communication to meet patients’ informational needs were identified as highly important [21].

Our literature search revealed only one randomized controlled trial which investigated a specific single episode information intervention in the ICU [22]. The investigators used audiotaped information after the patient’s recovery from anesthesia. The results of this study suggest the effectiveness of a single episode situational information intervention to improve psychological parameters in the ICU. The effectiveness of information administered face-to-face to ICU patients in the ICU has not yet been investigated.

Therefore the aim of our trial was to evaluate whether a structured information program modeled as a brief single episode nursing care delivery of ICU-specific information via face-to-face communication compared to a non-specific verbal face-to-face communication of the same length contributes to a reduction of experienced anxiety in ICU-patients. Both interventions were supplementary to standard care and administered in the initial stage of treatment in an ICU [23].

## Methods

### Trial design

We conducted a prospective, multi-center, non-blinded, randomized controlled trial with two parallel groups in a 1:1 ratio. The study was registered with ClinicalTrials.gov as NCT00764933. Informed consent was obtained from all patients before inclusion in the study. The trial is reported in accordance with the CONSORT statement [24].

### Participants

The study was performed between December 2007 and December 2009 in three hospitals in Germany (Marburg (coordinator) Halle, and Stuttgart) in cardiac surgery, general surgery, and medical ICUs. The study protocol [23] was approved by the respective ethics committees responsible for the study sites in the universities in Marburg, Halle, and Tübingen (Germany).

ICU patients in cardiac surgery, general surgery, and internal medicine (including local High Dependency Units) with scheduled and unscheduled ICU stays longer than 24 hours from enrollment were eligible for inclusion in our trial. All patients were recruited at the beginning of their ICU stay (meaning within the first 24 hours of consciousness post-admission). Exclusion criteria were: anticipated inability to fill in the mailed follow-up questionnaire, cognitive impairment, lack of German language ability, placed in a room with another study patient, more than 48 hours awake and clear in the ICU (because intervention was intended at the beginning of the ICU stay), or under the age of 18.

Study nurses assessed cognitive impairment by means of the Richmond Agitation and Sedation Scale (RASS) [25] and the Confusion Assessment Method for the Intensive Care Unit (CAM-ICU) [26-28]. RASS values < −3 or a positive test of CAM-ICU (acute confusion: yes) were deemed as an inability to consent to study participation on the day of the assessment. Study nurses assessed these patients again on the following day. In this way it was possible for patients to be assessed more than once for study inclusion (temporary exclusion when patient was not able to give consent due to impaired consciousness). We only report the last-encountered reason for the exclusion of these patients.

### Interventions

Effects of information interventions are often explained by the provision of individual relevant information and psychosocial support, making the situation more understandable and bearable for the patient. These explanations are concordant with Lazarus’ cognitive-mediation theory of stress and emotion [29] which emphasizes the role of cognitive aspects (appraisal-reappraisal, emotion-focused coping, and emotional social support, tangible social support and informational social support) in the development of anxiety and stress. This framework also seems well-suited for an intervention rationale that encompasses giving information to all patients admitted to ICUs (i.e. including non-elective surgical patients or non-surgical patients). Therefore our study intervention applied Lazarus’ cognitive-mediation theory of stress and emotion.

The experimental intervention was a single episode information intervention that included ICU-specific information and was implemented at an early stage of the patients’ ICU stay to intensify information given in standard care process. We decided to develop and implement the experimental intervention as single episode information for two reasons: (1) The intervention should be pragmatic in regard to applicability and implementation fidelity. (2) Studies in regard to pre-operative information [30,31] and Hwang et al. [22] successfully used single episode interventions. The intervention was designed as a guided conversation with both a standardized and an individualized part (Table 1). The standardized part of the intervention covered general information on nine topics (Table 2), which have been identified as relevant for patients in ICUs in previous studies [32-34]. All patients in the intervention group (IG) received this level of information. In the individual part the patients were allowed to choose any number of cards from seven that depicted common fears (Table 1). Complementary, the patients’ needs for personal information on particular topics of the first part or additional topics were assessed by asking the patients if they wanted more detailed information of certain topics. These topics and the chosen cards (i.e. fears) were addressed in the subsequent individualized conversation. So IG patients had the opportunity to get sensory, procedural, and coping information on ICU-specific and additional topics, and could speak about their fears and anxieties.

**Table 1:** Structure of the experimental intervention and justification of its components

**Part of the intervention**	**Description**	**Justification**
I. Standardized part	General information on nine relevant topics in the ICU using a guideline (with examples for each topic)	Topics were identified in trials of pre-operative information and patient education. A guideline was chosen to achieve a standardized structure but still having the possibility to make setting and ICU specific adaptations.
II. Individualized part	Seven cards the patient could choose from, depicting common fears associated with an ICU-stay:	The cards offered patients the opportunity to start a conversation about specific fears, by helping them to articulate fear. So it was possible for the trial staff to answer accordingly (e.g., What is being done in the ICU to prevent this from happening to you?). Additionally vocally impaired patients (e.g. by mechanical ventilation) could indicate their fears, too. The opportunity to ask further questions was to meet informational needs in patients that wanted to know more or more detailed about their ICU stay.
	1. fear of complications	
	2. fear of suffocating	
	3. fear of pain	
	4. fear of being helpless	
	5. fear of death	
	6. fear of being lonely	
	7. fear of being confined	
	Opportunity to ask additional and detailed questions on the ICU stay (recurring to the topics of part I or additional topics)	

**Table 2:** Topics of the standardized part of the experimental intervention

**Nr.**	**Topic**	**Details**
**1**	**People in the ICU**	• health care professionals (nurses and intensive care nurses)
		• attending physician
		• clothing, including specifics such as masks, gloves etc.
		• change of shifts
		• ward rounds
**2**	**Devices and monitoring**	• monitor, including central monitoring
		• ventilator
		• infusion and syringe pump
		• alarms
**3**	**Room furnishing**	• clock
		• bell system
		• room size
**4**	**Individual safety**	• tubes, drainages, wounds, urinary catheters, fixation
		• tube, respiratory mask, mechanical ventilation
		• waking phase
		• intravenous access
		• bedding
		• dimming of the light
**5**	**Schedule**	• hospital stay duration
		• transfer to IMC
		• differences between IMC and ICU
		• nutrition
**6**	**Communication**	• nod, shake of the head (yes, no)
		• pens
**7**	**Staff duties**	• aspiration
		• mobilization
		• radiologic examinations
		• personal hygiene/oral hygiene
**8**	**Conveniences**	• pain medication
		• visiting hours
		• information before nursing-medical interventions
**9**	**Helpful thoughts**	• “Everything is done for me. That is a sign that everything worked alright”.
		• “I don’t have to suffer from any pain; if necessary I will receive additional medication. In the meantime I can relax and continue to breathe calmly”.
		• “Only a little longer, then I have made it”.

The study investigators that were responsible for intervention development were all experienced within the field of intensive and anesthesia nursing care or clinical psychology. The nine topics and the seven fears on the cards were chosen in accordance with the research literature on pre-operative information and relieving aspects of ICU stay, and our own preliminary studies [34].

The control intervention was a non-specific conversation of the same length and was offered at the beginning of ICU stay as a semi-structured, self-directed, non-specific conversation (excluding information on the ICU stay) with a study nurse. Priority was given to topics that were related to overall health, family, occupational concerns, and recreation.

Both interventions were piloted in each study center to test feasibility and acceptability of both interventions.

A study nurse carried out the study procedure in each center, but none of the study nurses were involved in the standard care of the included patients. Treatment fidelity in the different centers was intended by comprehensive instruction of the study personnel for the implementation of the information and the control intervention. Study nurses documented length and content of the information and the control intervention. Thus, assessment of fidelity was done on a descriptive indirect level only. Additionally, the coordinating center in Marburg centrally monitored the trial by regular visits and clinical supervision in the participating study centers.

Both interventions took place immediately after recruitment, baseline measurement, and randomization and were supplementary to standard care.

### Outcomes

All measurements and the timeline are summarized in Table 3.

**Table 3:** **Measures in the course of the study, including baseline data (t**_**0**_**) and outcomes (t**_**1 **_**– t**_**5**_**)**

	**Point of measurement**	**Measurements**
t_0_	Day of enrolment (ICU) (before randomization)	Socio-demographic data, routine treatment data, acute confusion (CAM-ICU including RASS), anxiety state (FAS)

t_1_	24 h after study intervention	Acute confusion (CAM-ICU including RASS), anxiety state (FAS)
t_2_	48 h after study intervention	Acute confusion (CAM-ICU including RASS), anxiety state (FAS)
t_3_	Admission to regular ward	ICU related anxiety and experiences (CINT questionnaire including CINT-Score), anxiety state (VAS-A, and STAI-State)
t_4_	Discharge from hospital	In-patient history and complications, length of stay and mode of discharge
t_5_	3 months after discharge (via post)	Individual quality of life (SEIQoL), health related quality of life (SF-12)

#### Primary outcome

The primary outcome was the anxiety-related part (CINT-Score) of the Questionnaire for Surgical ICU Patients (CINT questionnaire) on the experiences and the emotional state in the ICU that was recorded shortly after admission to a regular ward. This questionnaire has already been used in pilot studies [32,33,35] and represents specific aspects of quality of life in relation to the ICU.

The CINT-Score covers experienced anxieties during the ICU stay and comprises the following items: fear of death, fear of severe suffering, fear of a handicap, fear of the future, fear of uncertainty, panic, strain, depression, loneliness, melancholy, lack of orientation, uncertainty, anger, optimism, and confidence. All items were rated on a 4-point scale from *never* to *always*. The final score was calculated as a Likert-scale, averaging the items (after inverting negative items) and transforming it to a scale of 0–100 (no anxiety to maximum anxiety). In an earlier study [35] the CINT-Score has shown good internal consistency (Cronbach’s alpha = 0.88) (unpublished data).

#### Secondary outcomes

In addition to the retrospective CINT-Score a visual analogue scale supported by the Faces Anxiety Scale (FAS) [36] was used for measurements at 24 h and 48 h after intervention (ranges from 0–100; no anxiety to maximum anxiety). The FAS has shown an acceptable level of validity in the ICU setting even for ventilated patients, while having a low respondent burden [36-39]. As a graphic representational scale, reliability can be assumed, too [40]. Additionally, the patients’ level of consciousness and concentration regarding a potential ICU-related state of confusion (acute confusion: yes/no) was assessed with the CAM-ICU [26,27], including the RASS [25]. The CAM-ICU demonstrated good validity and reliability in ICU patients [41].

To compare anxiety levels after the ICU stay, the State Scale of the State and Trait Anxiety Inventory (STAI) [42] was recorded and calculated in accordance with the manual (ranges from 20–80). The STAI-State is a well-established measurement for anxiety with good validity, reliability, and has shown responsiveness to change [42]. This measure was transferred to a 0–100 scale (no anxiety to maximum anxiety). The Visual Analogue Scale-Anxiety (VAS-A) [43] (ranges from 0–100; no anxiety to maximum anxiety) was measured in parallel to the STAI-State. VAS-A has shown validity but data on reliability is limited [43].

ICU-related experiences were obtained with the CINT questionnaire (36 single items in relation to the categories: communication, physical state, environmental factors, and ICU-specific circumstances). For the analysis of additional effects the length of stay (ICU and hospital) were extracted from patients’ records.

Three months after discharge a questionnaire was mailed to the participants of both study groups. We asked about health-related quality of life (QoL) using the Health Survey 12 Item Short Form (SF-12) [44], and individual QoL using the Schedule for Evaluation of Individual Quality of Life (SEIQoL) [45]. For this reason we developed and implemented a paper questionnaire version of the SEIQoL [46] which was used and tested for feasibility the first time in our study. SF-12 results were analyzed in accordance with the manual in the dimensions of the Mental Health Component Summary (SF-12 MCS) score and the Physical Health Component Summary (SF-12 PCS) score. The SF-12 has shown good reliability and validity [47]. All QoL scores range from 0–100 (higher scores indicate better QoL).

### Sample size

The sample size calculation was based on the CINT-Score as an anxiety related aspect of the ICU-patients’ quality of life. This sum score is represented on a scale from 0 to 100 and was found to add up to a mean value of M = 28.0 and a standard deviation of SD = 17.0 in an earlier unpublished trial in an ICU population. On the notion that in QoL measures the differences within the scope of half a standard deviation are considered as noticeable [48] we determined a difference of 8.5 scale points to be clinically relevant. Further determining α = 0.05 and β = 0.20, 70 patients per group were needed to find this effect from a standard two-group *t*-test. The allowance of a 30% loss to follow-up resulted in an estimated sample size of n = 100 per group, corresponding to a total sample size of N = 200 (n = 100 vs. n = 100).

### Randomization

A statistician computer generated the randomization sequence in advance employing a stratified (by center and clinical department) balanced block randomization. A study nurse employed for the trial randomized participants individually. Group allocation was concealed by using sequentially numbered opaque sealed envelopes and took place right after informed consent was received and baseline assessment. Contamination was avoided by restricting recruitment to one patient per room in the ICU at the same time.

### Statistical methods

All data was double entered using double entry verification provided by EpiData Data Entry [49]. Data analysis was performed using the statistical software R [50]. Final data analysis was done in accordance with the published study protocol [23].

Analysis of the primary endpoint used an ANCOVA model with the primary endpoint as the response, and the center as a covariate, thus allowing for the stratified randomization procedure. The treatment effect was calculated as an adjusted difference in means with a corresponding 95% confidence interval (CI 95%), α was set to 0.05.

Secondary endpoints were assessed with the respective adjusted models, using standard ANCOVA models for continuous outcomes. Mixed effects models were used for repeatedly measured outcomes to adjust for within-patient correlations [51]. Secondary endpoints analyses were considered as merely exploratory to avoid alpha-inflation.

The analyses of all outcomes were by intention to treat (all patients analyzed as they were randomized). However, no imputation of missing data was performed, and we did complete cases analyses. Missing data for single measures was handled in accordance with the respective measure’s manual.

## Results

**Figure 1:**
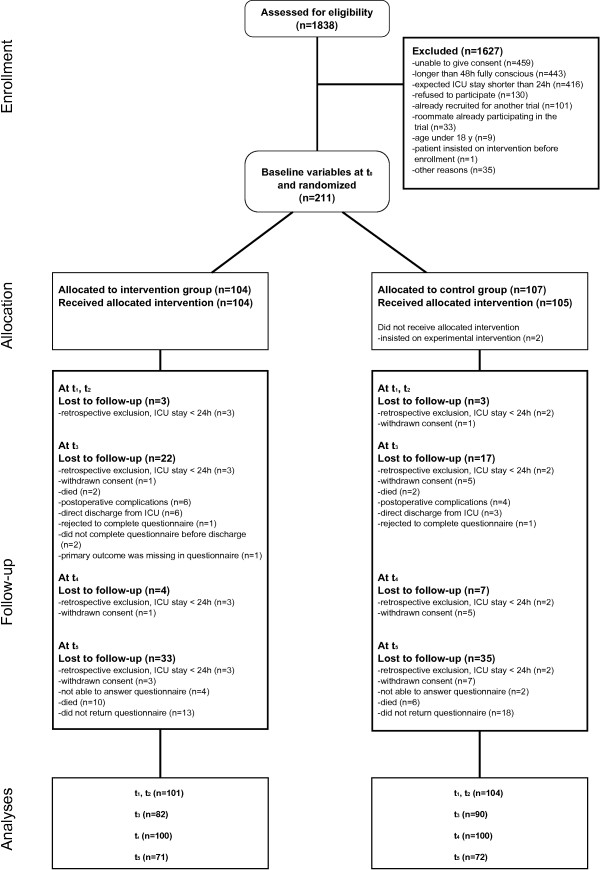
Flow of participants through the trial.

A total of 1838 ICU patients were assessed for eligibility. Figure 1 displays the reasons for study exclusion. The three main reasons for study exclusion were *unable to give consent*, *longer than 48 h fully conscious*, and *expected ICU stay shorter than 24 h*. We recruited 211 patients with 92 patients in Marburg, 59 patients in Halle, and 60 patients in Stuttgart. Randomization resulted in 104 intervention group participants and 107 control group participants. Two patients in the control group received the information intervention as they insisted on it after randomization, but were analyzed in the control group.

Baseline characteristics are displayed in Table 4. There were no clinically relevant differences at baseline except for baseline anxiety measured with the FAS. We conducted sensitivity analyses controlling for FAS at t_0_ and compared these analyses with our predefined analyses.

**Table 4:** Baseline characteristics of the study population

**Variable**	**Intervention group (n = 104)**	**Control group (n = 107)**
Sex, n (%)		
Men	66 (63.5)	71 (66.4)
Women	38 (36.5)	36 (33.6)
Age, years		
Mean (SD)	63.3 (14.5)	65.8 (11.8)
Median (IQR)	68.0 (55.0-73.0)	68.0 (59.0-74.0)
Department, n (%)		
Cardiac surgery patients	52 (50.0)	50 (46.7)
General surgery patients	19 (18.3)	22 (20.6)
Medical patients	33 (31.7)	35 (32.7)
Main procedure, n (%)		
Surgical intervention	71 (68.3)	79 (73.8)
Non-surgical intervention	21 (20.2)	22 (20.6)
Diagnostic measure	10 (9.6)	3 (2.8)
Other procedure	2 (1.9)	3 (2.8)
ICD-10 chapter, main diagnosis, n (%)		
Diseases of the circulatory system	70 (67.3)	82 (76.6)
Neoplasms	5 (4.8)	8 (7.5)
Diseases of the digestive system	5 (4.8)	6 (5.6)
Diseases of the respiratory system	7 (6.7)	1 (0.9)
Injury, poisoning and certain other consequences of external causes	4 (3.8)	4 (3.7)
Congenital malformations, deformations, and chromosomal abnormalities	4 (3.8)	1 (0.9)
Other chapters^*a*^	9 (8.7)	5 (4.7)
FAS^*b*^ (t_0_)		
Mean (SD)	24.5 (18.9)	30.2 (22.1)
Median (IQR)	20.0 (10.0-37.5)	25.0 (10.0-48.8)
SAPS II^*c*^ (t_0_)		
Mean (SD)	23.8 (8.2)	26.1 (10.2)
Median (IQR)	23.0 (18.0-29.0)	24.5 (20.0-31.0)
TISS 28^*d*^ (t_0_)		
Mean (SD)	10.6 (5.6)	11.5 (6.5)
Median (IQR)	10.0 (5.0-14.0)	10.0 (10.0-14.0)
Type of ventilation in intensive care unit, n (%)		
No ventilation	34 (32.7)	40 (37.4)
Non-invasive ventilation	8 (7.7)	2 (1.9)
Invasive ventilation	62 (59.6)	65 (60.7)
Ventilation in intensive care unit before the intervention, hours^*e*^		
Mean (SD)	25.0 (46.6)	41.8 (127.2)
Median (IQR)	10.3 (6.0-18.6)	10.0 (7.0-20.0)

^*a*^Summarizes all other ICD-10 chapters with less than five patients in total; ^*b*^ranges from 0 (no anxiety) to 100 (maximal anxiety); ^*c*^higher score means worse overall health; ^*d*^higher score means higher intervention needs; ^*e*^only ventilated patients, before interventions.

### Intervention fidelity

Data on implementation of the interventions in both groups are presented in Table 5. Intervention length differed significantly between the two study groups (IG mean: 13.5 minutes (SD 3.6) vs. CG mean 11.3 minutes (SD 3.9), mean difference = 2.2 minutes, CI 95% (1.2; 3.2), p < 0.001).

**Table 5:** Circumstances under which the interventions were applied

**Variable**	**Intervention group (n = 104)**	**Control group (n = 107)**
Duration of intervention, minutes		
Mean (SD)	13.5 (3.6)	11.3 (3.9)
Median (IQR)	15.0 (10.0-15.0)	10.0 (10.0-14.0)
Day of intensive care unit stay		
Mean (SD)	1.1 (1.6)	1.7 (3.1)
Median (IQR)	1.0 (0.0-1.0)	1.0 (1.0-1.0)
Ventilated during intervention, n (%)		
Yes	5 (4.8)	7 (6.5)
No	99 (95.2)	100 (93.5)
Family members present, n (%)		
Yes	5 (4.8)	3 (2.8)
No	99 (95.2)	104 (97.2)

Of the patients that received the experimental intervention (n = 106, IG patients plus the two CG patients that received the experimental intervention), 61 patients (57.5%) chose at least one fear-depicting card presented to them. Overall 124 cards were drawn (multiple cards per person were possible). The three most frequent fears were: *fear of complications* (n = 34 times drawn), *fear of suffocating* (n = 27 times drawn), and *fear of pain* (n = 25 times drawn). Forty-five patients (42.5%) did not choose any of the cards presented to them. In regard to the ICU-specific topics, 74 patients (69.8%) that received the experimental intervention (n = 106, IG patients plus the two CG patients that received the experimental intervention) asked for more detailed information on the topics of the first conversation part and/or other topics: resulting in 327 requests on the topics; multiple requests per person possible. The three most frequent topics were: *devices and monitoring* (n = 55 times asked), *room furnishing* (n = 47 times asked), and *people in the ICU* (n = 44 times asked). Thirty-two patients (30.2%) did not ask for further information on the topics of the first conversation part or additional topics.

The main conversation topics (only the main topic of conversation was documented) in the control group (n = 105, CG patients minus the two CG patients that received the experimental intervention) were: *overall health status* (n = 40), *family* (n = 39), *recreation* (n = 11), *occupation* (n = 11), and *other topics* (n = 4).

### Follow-up

For analyses of repeated measurements (t_1_, t_2_) 205 patients (97%) were eligible. One-hundred and seventy-two patients (82%) could be included for the analysis of the primary outcome (t_3_). The mailed final questionnaire was answered by 143 patients (68%) (t_5_). Reasons for incomplete data are presented in Figure 1. There were no differences, either in numbers or reasons for loss to follow-up, between the two study groups.

In order to check similarity of IG and CG after drop out we compared baseline values of the complete cases for the primary outcome. Overall patients with a higher FAS, SAPS II, and TISS 28 were more likely to be lost to follow-up in our sample but characteristics still were evenly distributed between the IG and the CG.

### Outcomes

**Figure 2:**
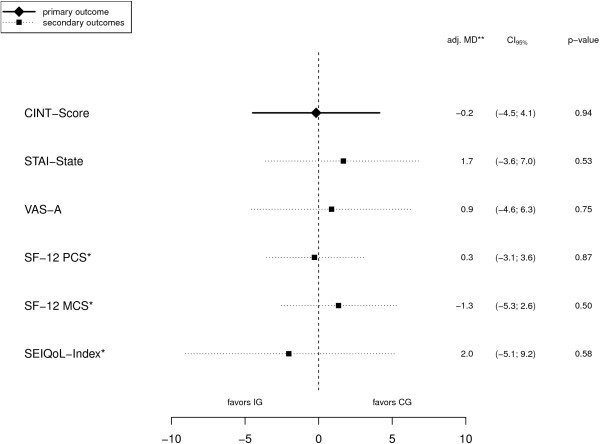
Treatment effects for primary and secondary outcomes (* values are inverted in the graphical display; ** adjusted for study center).

Adjusted mean differences (adj. MD) and related 95% CIs are presented in Figure 2. There were no significant and no clinical relevant differences between the two groups for the primary and secondary outcomes. A sensitivity analysis with FAS t_0_ as an additional covariate did not show different results; therefore only results from the model with study center as a covariate are presented. The raw means without adjustment are presented in Table 6.

**Table 6:** Results of the primary and secondary outcomes

	**IG Mean (SD)**	**CG Mean (SD)**
t_3_ measurements: admission to regular ward		
CINT-Score^*a*^	20.4 (14.4)	20.8 (14.7)
STAI-State (transformed)^*a*^	33.0 (17.0)	32.1 (19.1)
STAI-State (original)^*b*^	40.0 (10.4)	39.2 (11.1)
VAS-A^*a*^	12.7 (18.6)	11.9 (17.4)
t_5_ measurements: 3 months after discharge		
SF-12 PCS^*c*^	40.6 (9.4)	40.4 (10.0)
SF-12 MCS^*c*^	46.9 (11.3)	48.2 (11.2)
SEIQoL-Index^*c*^	74.9 (18.2)	73.6 (20.1)

Cases with complete data: CINT-Score (IG/CG 82/90); STAI-State (79/89); VAS-A (81/88); SF-12 PCS (64/66); SF-12 MCS (64/66); SEIQoL-Index (58/60); ^*a*^ranges from 0 (no anxiety) to 100 (maximum anxiety); ^*b*^ranges from 20 (no anxiety) to 80 (maximum anxiety); ^*c*^ranges from 0 to 100, higher scores mean higher quality of life.

Values are unadjusted mean values (standard deviation).

The treatment effect as a mean difference with regard to the repeated measurement of the FAS (t_0_ through t_2_) was calculated as a fixed effect of a mixed effects model with a random intercept. Sensitivity analyses with and without FAS t_0_ as a covariate showed considerable influence of baseline anxiety. The fixed effect was −7.8, 95% CI −15.7 to 0.1, p = 0.05, in favor of the IG when not controlling for FAS t_0_. This effect changed to −2.4, 95% CI −6.4 to 1.5, p = 0.14, in favor of the IG when controlling for FAS t_0_. Thus, our preplanned analysis (Model 1) for this secondary outcome does not safely exclude the possibility of a clinically relevant difference between the two groups while an analysis that controls for baseline FAS (Model 2) and its related CI 95% makes a clinically relevant difference in future research rather unlikely. Therefore we favor Model 2 as it is more conservative to the null hypothesis.

In regard to the prevalence of acute confusion in the ICU assessed with the CAM-ICU (t_0_ through t_2_) a mixed effects logistic regression model (Generalized Linear Mixed Model with Penalized Quasi-Likelihood Estimation) using our basic model did not converge. Therefore we used a binomial Generalized Linear Model without random effects to get an impression of intervention effects. The comparison of IG vs. CG revealed an odds ratio of 0.33, 95% CI 0.05 to 1.49, p = 0.18, to suffer from acute confusion in favor of the IG (t_0_ IG: 0/104, CG: 0/107; t_1_ IG: 2/96, CG: 6/100; t_2_ IG: 0/70, CG: 5/72).

Mean length of ICU stay was 4.3 (SD 4.5) days in the IG vs. 4.9 (SD 5.5) days in the CG with a mean difference adjusted for study center of −0.4 days in favor of the IG, 95% CI −1.7 to 0.9, p = 0.56. Overall mean length of hospital stay was 15.4 (SD 13.8) days in the IG vs. 16.5 (SD 13.5) days in the CG with a mean difference adjusted for study center of −1.0 days in favor of the IG, 95% CI −4.7 to 2.7, p = 0.59.

On the item level of the CINT-Score and the CINT questionnaire no significant group differences could be found. We did not predefine any adverse events as we did not expect negative effects of both study interventions.

## Discussion

This is the first trial that tested a personal single episode information intervention in ICU patients. Our trial failed to demonstrate a significant decrease of anxiety measured with the CINT-Score in the IG. We found no benefit for an ICU-specific single episode information intervention in the ICU compared to an unspecific conversation of comparable length. This result is the opposite of former studies that investigated single episode information given preoperatively [30,31] or to the findings of Hwang et al. [22]. For some secondary outcomes (length of ICU stay, length of hospital stay, and prevalence of acute confusion) there may be a signal for improvement. However these improvements must be interpreted with caution as there was too much statistical uncertainty.

Our study had certain limitations. We cannot exclude a reduced effect of an information intervention due to patients’ limited memory, and thus information processing ability [52]. However, our recruitment and screening procedure by means of RASS and CAM-ICU most likely lead to an underrepresentation of confused or cognitively impaired patients in our sample. Study personnel and participants were not blinded to allocation after randomization. Although we do not expect a high risk of performance bias since the investigators were not involved in patient care and co-interventions can be excluded. Measures of treatment effect used in this study mainly included patient reported outcomes and no physiologic measures like psycho-endocrine stress indicators, which are highly associated with anxiety [53]. Psycho-endocrine stress indicators may represent a more objective method to measure emotional distress than a one-item scale like the FAS or a retrospective questionnaire like the CINT-Score. However, the validity of these indicators could be compromised by applied drugs and health status of the participants in the ICU [54]. FAS and CINT-Score values indicate rather low levels of anxiety in our study sample (Table 6). This corresponds to the high proportion (42.5%) of patients that did not select a fear card in the IG, indicating they saw no need for further communication on fears. A further explanation could be the recruitment procedure, as our sample had an overrepresentation of “healthier” ICU-patients than is generally found in patients in high dependency units or intermediate care wards. Consequently, a reduction of an already low level of anxiety seems rather unlikely. It has to be questioned whether anxiety is a relevant outcome for information interventions in cognitive unimpaired ICU patients or if stereotyped information actually can have positive effects compared to more patient centered communication approaches. Nevertheless, a certain proportion of ICU patients suffered considerably from fear and anxiety. Informational needs seem to vary widely as indicated by the large variability of chosen cards and requested information topics in our study. Approaches that encompass more continuous information giving and patient centered communication, i.e. interventions like staff education on communication and patient information may be more promising to investigate.

The measurement of the Therapeutic Intervention Scoring System (TISS 28) [55] and Simplified Acute Physiology Score (SAPS II) [56] at baseline indicate that our study population compares well to ICU patients in other studies. While the mean TISS 28 in the original validation study [55] was substantially higher than in our study, meaning we had included a sample with considerably fewer care activities than the former validation sample. The same holds true for the severity of illness measured with the SAPS II. Most studies that used the SAPS II [57-59] showed a higher mean score (i.e. higher severity of illness) than our study sample. The lower TISS 28 and SAPS II scores of our study are correspond with the low ICU related mortality (mortality rate until t_3_) in our study sample (IG: 2/104; CG: 2/107). These indicators for a positively selected sample from the ICU population are further reinforced by the low prevalence of patients with acute confusion compared to 17% for cardiac surgery patients in other studies [60]. This overall positive selection of our study sample can mainly be explained by our inclusion criteria, specifically due to the absence of a cognitive impairment as a prerequisite for informed consent and enrolment in our study.

Our study had also strengths. To avoid an intervention effect simply due to increased personal attention we implemented optimized usual care through unspecific conversation of the same length as a control intervention instead of a null intervention. Thus, the comparator investigated in our study is more rigorous than the one used in the study by Hwang et al. [22] where an intervention effect due to increased attention cannot be safely excluded. Although the length of the two interventions in the IG and CG differed significantly we rated the difference as clinically none-relevant. As indicated by Table 5, we were able to achieve good fidelity to our study protocol [23] for both groups and to perform the interventions in an early stage of the ICU stay. In regard to a difficult study population we were able to achieve our preplanned sample size. We published our study protocol and registered our study. No deviation from our study protocol occurred.

## Conclusions

We conclude that a single episode information intervention in the ICU has no benefit to cognitively unimpaired patients with a low intervention profile (i.e. low scores in TISS 28 and SAPS II) compared to a nonspecific personal conversation. Therefore, single episode structured information for patients early in their ICU stay cannot be recommended for routine use. Approaches that try to improve staff communication skills in general and provide information during the whole ICU stay should be investigated in future ICU communication and information research.

### Key messages

•A single episode information intervention during ICU stay does not reduce anxiety in patients compared to an unspecific conversation of similar length.

•More intensified approaches that emphasize improvement of nurse-patient communication and information throughout the entire stay might be more successful and should be targeted in future studies.

## Abbreviations

ANCOVA: Analysis of covariance; CAM-ICU: Confusion assessment method for the intensive care unit; CG: Control group; CINT: Fragebogen chirurgische intensivstation (*questionnaire for surgical ICU patients)*; CINT-Score: Anxiety-related part of the CINT; FAS: Faces anxiety scale; ICU: Intensive care unit; IG: Intervention group; M: Mean; QoL: Quality of life; RASS: Richmond agitation and sedation scale; SAPS II: Simplified acute physiology score; SD: Standard deviation; SEIQoL: Schedule for evaluation of Individual quality of life; SF-12: Health survey 12 item short form; SF-12 MCS: Mental health component summary of the SF-12; SF-12 PCS: Physical health component summary; STAI: State and trait anxiety inventory; TISS 28: Therapeutic intervention scoring system; VAS-A: Visual analogue scale-anxiety.

## Competing interests

The authors declare that they have no competing interests.

## Authors’ contributions

SF wrote the first draft of the manuscript. All authors contributed equally to the final manuscript and made substantial intellectual contributions. AB, SF, and TRN had the idea for the trial rationale and initiated the trial proposal. All authors were responsible for conception and design of the final study protocol. TRN was the responsible coordinator of the study center in Marburg, AH in Stuttgart, and JB in Halle. RB monitored all participating study centers in regard to compliance to the study protocol. RB, TRN, AH, SF, and AB developed the study intervention. OK planned the statistical analysis and randomization. RB and SF supervised data entry. OK and SF conducted the statistical analysis. All authors read and approved the final manuscript.

## Pre-publication history

The pre-publication history for this paper can be accessed here:

http://www.biomedcentral.com/1471-2253/14/48/prepub
